# Impact of selective reporting of antibiotic susceptibility testing results on meropenem prescriptions for the treatment of *Pseudomonas aeruginosa* infections after 2020 EUCAST criteria update: an observational study in a university hospital

**DOI:** 10.1186/s13756-022-01203-x

**Published:** 2022-12-30

**Authors:** Aline Munting, José Damas, Benjamin Viala, Guy Prod’hom, Benoit Guery, Laurence Senn

**Affiliations:** 1 Service of Infectious Diseases, Lausanne University Hospital and University of Lausanne, Lausanne, Switzerland; 2 Infectious Diseases Department, Alpes Leman Hospital, Contamine sur Arve, France; 3 Institute of Microbiology, Lausanne University Hospital and University of Lausanne, Lausanne, Switzerland; 4 Infection Prevention and Control Unit, Lausanne University Hospital and University of Lausanne, Lausanne, Switzerland

**Keywords:** Antibiotic stewardship, *Pseudomonas aeruginosa*, Meropenem prescriptions, EUCAST breakpoints table

## Abstract

**Background:**

We previously reported an increase in meropenem prescriptions for *Pseudomonas aeruginosa* infections in our hospital after the implementation of the 10th version of the EUCAST breakpoints table for *P. aeruginosa* in January 2020. As a consequence, antibiotic susceptibility testing results were adapted by masking meropenem for *P. aeruginosa* isolates susceptible to either ceftazidime, cefepime or piperacillin-tazobactam. We aimed to assess the changes in meropenem prescriptions after the implementation of the selective reporting.

**Methods:**

In this retrospective single-centre observational study, we analysed antimicrobial therapies prescribed for *P. aeruginosa* infections after the susceptibility testing results have been made available over three periods: “before EUCAST update”, “after EUCAST update without selective reporting” and “after EUCAST update with selective reporting”, at Lausanne University Hospital, Switzerland. We collected epidemiological, microbiological and clinical data. The primary outcome was the prescription of meropenem to treat *P. aeruginosa* infections after the release of susceptibility testing results. Secondary outcomes were the use of increased dosage of non-meropenem anti-pseudomonal drugs, and IDs’ consultations rates after the release of susceptibility testing results.

**Results:**

Among the 457 patients included, 65 (14.2%) received meropenem: 5/148 (3.4%) before EUCAST update, 51/202 (25.3%) after EUCAST update without selective reporting, and 9/107 (8.4%) after EUCAST update with selective reporting. Supervision and counselling from IDs as well as the use of increased dosages of non-carbapenem antibiotics increased in both periods after EUCAST update, compared to the first period, respectively: 40.5% (60/148) versus 61.4% (124/202) versus 51.4% (55/107) (*P* < *0.001),* and 57.9% (84/148) versus 91.1% (183/202) versus 90.7% (97/107) (*P* < *0.001)*.

**Conclusions:**

Selective reporting of antibiotic susceptibility testing results might decrease unnecessary meropenem prescriptions for the treatment of *P. aeruginosa* infections and could be part of multimodal antibiotic stewardship interventions.

**Supplementary Information:**

The online version contains supplementary material available at 10.1186/s13756-022-01203-x.

## Background

In January 2019, the 10th version of breakpoints table for *Pseudomonas aeruginosa* was updated by the European Committee on Antimicrobial Susceptibility Testing (EUCAST) [1]. As a result, wild-type *P. aeruginosa* isolates previously labelled “S” were labelled “I” for ceftazidime, cefepime and piperacillin-tazobactam, but remained “S” for meropenem. Since the implementation in our hospital of the new EUCAST criteria in January 2020, we reported increased odds of meropenem prescriptions [2].

Following this observation, the internal antibiotic stewardship committee decided to change the report of antimicrobial susceptibility testing results using the “selective reporting”, also known as “cascade reporting”, an antibiotic stewardship strategy of reporting susceptibilities of broad-spectrum agents only when the isolate is resistant to more narrow-spectrum agents [3–5]. Practically, since December 21, 2020, meropenem is no longer reported for patients with a *P. aeruginosa* isolate that is susceptible (“I”—“susceptible, increased exposure”) to at least one beta-lactam among ceftazidime, cefepime and piperacillin-tazobactam. However, if the isolate is resistant to meropenem, it is reported on the susceptibility testing results. Of note, clinicians can always ask the microbiology department to unmask meropenem if needed, in particular in polymicrobial infections or in case of allergy.

In the present study, we aimed to evaluate the impact of this selective reporting on meropenem prescriptions for *Pseudomonas aeruginosa* infections at Lausanne University Hospital.

## Methodology

### Study design and setting

This study is the continuation of a first study that took place at Lausanne University Hospital, a 1500-bed tertiary university hospital in Lausanne, Switzerland. The study setting has been previously described in details elsewhere [2].

### Study design and participants

We conducted a retrospective observational single-center study. All consecutive adult patients with *P. aeruginosa* isolated from a clinical sample between 01.08.2019 and 31.07.2021 were identified. Those who received an antibiotic for a *P. aeruginosa* infection and that could be treated either by ceftazidime, cefepime and/or piperacillin-tazobactam based on susceptibility testing results available in the Electronic Medical Record (EMR) were included in the study. We excluded patients with a *P. aeruginosa* isolate resistant to meropenem, and those with a *P. aeruginosa* infection that could not be treated by ceftazidime, cefepime or piperacillin-tazobactam due to allergy. We also excluded patients with a polymicrobial infection requiring a treatment with a carbapenem, including ESBL-producing *Enterobacterales* co-infections.

Three periods were defined: the first from 01.08.2019 to 26.01.2020-patients treated “before EUCAST update” (period 1), the second from 27.01.2020 to 20.12.2020—patients treated “after EUCAST update without selective reporting” (period 2) and the third from 21.12.2020 to 31.07.2021—patients treated “after EUCAST update with selective reporting” (period 3).

### Data collection

Epidemiological, clinical and microbiological data were extracted from the EMR. Data collection for patients meeting the same criteria from 01.08.2019 to 31.07.2020 had been done in the previous study [2]. Epidemiological data included age, sex, and relevant comorbidities. We also collected data on microbiology results, antimicrobial therapy, stay in intensive care, infectious diseases specialist (IDs) consultations, and other clinical aspects: site and severity of infection, community versus healthcare-associated infection—including vascular catheter-associated infections, catheter-associated urinary tract infections, ventilator-associated pneumonia, surgical site infections and infections occurring more than 48 h after admission to hospital. We entered all the data in an electronic clinical report form (eCRF) using the Redcap® platform (Research Electronic Data Capture v10.3.3, Vanderbilt University, Tennessee, USA).

In case of multiple episodes of *P. aeruginosa* infection, patients included a first time before the implementation of selective reporting could be included once again after selective reporting.

### Outcomes

The primary outcome was meropenem prescription as targeted treatment (*i.e.* after *P. aeruginosa* susceptibility testing release). We took into consideration the antipseudomonal antibiotic initiated after the susceptibility testing results have been made available. For patients receiving empiric antipseudomonal antibiotic therapy initiated before susceptibility testing results, we took into consideration the ongoing antipseudomonal antibiotic 24 h after the susceptibility testing results have been made available.

Secondary outcomes were the use of increased dosage for non-meropenem anti-pseudomonal drugs, and IDs consultation rates after susceptibility testing results have been made available.

### Statistics

For the descriptive analysis, we summarized categorical variables as numbers (percentages), normally distributed continuous variables as mean ± standard deviation (SD), and continuous variables with a skewed distribution as median [interquartile range (IQR)]. Between-group comparisons were performed using chi-square or Fisher’s exact test for qualitative variables, and Student’s t-test, analysis of variance or Kruskal–Wallis test for quantitative variables.

Analyses for our primary outcome were performed through uni- and multivariable logistic regression models. Models were built manually, adding demographic, and clinical characteristics. We then added variables with a *P* value below < 0.2 from our univariable analysis. Models were built based on Akaike Information and Bayesian Information Criteria. We calculated Odds ratio (OR) with 95% confidence interval (95% CI) to determine the weight of risks factors for meropenem prescription. *P *values < 0.05 were considered as statistically significant. Due to the important number of dependent events and the small number of meropenem prescriptions, especially for period 1, these models did not allow us to perform a robust analysis. Therefore, we report only descriptive results. We used Stata SE 17.0 (StataCorp, College Station, TX) for all analyses.

## Results

Among 1329 patients with *P. aeruginosa* documented in a clinical sample between 01.08.2019 and 31.07.2021, 457 patients met inclusion criteria: 148 in the first period, 202 in the second period and 107 in the third period (Additional file 1: Supplementary figure 1). Demographic, clinical and microbiological characteristics of included patients are presented in Table 1.

**Table 1:** Demographic and clinical characteristics of patients

Variables	Overall (N = 457)	Before EUCAST update (n = 148)	After EUCAST update, without selective reporting (n = 202)	After EUCAST update, with selective reporting (n = 107)	*P*-value (all periods)	*P-* value (period 2 vs period 3)
**Female**	176 (38.5)	58 (39.2)	73 (36.1)	45 (42.1)	0.5	0.3
**Age**	68.7 (54.4; 78.2)	68.7 (51.5; 77.4)	69.8 (57.1; 78.9)	65.6 (53.3; 77.9)	0.8	0.4
Immunosuppression*	78 (17.1)	26 (17.6)	39 (19.3)	13 (12.15)	0.2	0.1
Immunosuppressive treatment	17 (3.7)	7 (4.7)	9 (4.5)	1 (0.9)	0.2	0.09
Myeloablative chemotherapy (< 1 month)	9 (1.9)	4 (2.7)	5 (2.5)	0 (0.0)	0.2	0.1
Neutropenia	17 (3.7)	3 (2.0)	12 (5.9)	2 (1.9)	0.08	0.1
Solid organ transplant recipient	21 (4.6)	8 (5.4)	11 (5.5)	2 (1.9)	0.3	0.1
Other Immunosuppression	29 (6.4)	10 (6.8)	13 (6.4)	6 (5.6)	0.8	0.7
History of ESBL producing Enterobacteriaceae (infection/colonisation during the six previous months)**	10 (2.2)	4 (2.7)	3 (1.5)	3 (2.8)	0.6	0.4
**Severity of current infection**					0.2	0.1
None	364 (80.0)	115 (77.7)	160 (79.2)	91 (85.1)		
Sepsis	63 (13.9)	25 (16.9)	25 (12.4)	13 (12.2)		
Sepsis shock	28 (6.2)	8 (5.4)	17 (8.4)	3 (2.8)		
**McCabe score for current infection**					**0.003**	**0.04**
Rapidly fatal disease (< 1 year)	69 (15.1)	32 (21.6)	30 (14.9)	7 (6.5)		
Ultimately fatal disease (1–4 years)	164 (35.9)	41 (27.7)	74 (36.1)	51 (46.7)		
Non-fatal disease (> 5 years)	224 (49.1)	75 (50.7)	99 (49.1)	50 (46.7)		
**Patients’ location**					0.1	0.2
Emergency Room	5 (1.1)	2 (1.4)	3 (1.5)	0 (0.0)		
Intensive care	56 (12.3)	14 (9.5)	28 (13.9)	14 (13.1)		
Medical ward	178 (38.9)	52 (35.1)	89 (43.8)	37 (34.6)		
Rehabilitation ward	9 (1.9)	2 (1.4)	3 (1.5)	4 (3.7)		
Surgical ward	154 (33.7)	53 (35.8)	60 (28.7)	41 (38.3)		
Outpatient	55 (12.1)	25 (16.9)	19 (9.4)	11 (10.3)		
**Infection’s setting**					**0.05**	**0.9**
Community acquired	201 (43.9)	77 (52.1)	81 (40.1)	43 (40.2)		
Healthcare-associated***	256 (56.1)	71 (47.9)	121 (59.9)	107 (59.8)		
Site of infection					0.2	0.4
Bacteraemia without focus	8 (1.7)	0 (0.0)	7 (3.5)	1 (0.9)		
Bone/joints	30 (6.5)	11 (7.4)	11 (5.5)	8 (7.5)		
Catheter-related bacteraemia	9 (1.9)	1 (0.7)	5 (2.5)	3 (2.8)		
Central nervous system	2 (0.4)	0 (0.0)	0 (0.0)	2 (1.9)		
Digestive	30 (6.6)	9 (6.1)	17 (7.9)	5 (4.7)		
Endovascular	6 (1.3)	4 (2.7)	2 (0.9)	0 (0.0)		
ENT	23 (5.1)	8 (5.4)	9 (4.5)	6 (5.6)		
Gynaecological/obstetrical	1 (0.2)	1 (0.7)	0 (0.0)	0 (0.0)		
Low respiratory tract	176 (38.5)	62 (41.9)	71 (35.2)	43 (40.2)		
Mucocutaneous	52 (11.4)	13 (8.8)	24 (11.9)	15 (14.2)		
Urinary tract	110 (24.1)	37 (25.0)	51 (25.3)	22 (20.6)		
Other soft tissue infection	10 (2.2)	2 (1.4)	6 (2.9)	2 (1.9)		
**Gram negative rod coinfection**	117 (25.6)	39 (26.4)	56 (27.7)	22 (20.6)	0.3	0.1
**Antibiotic prescription before susceptibility testing**^‡^	334 (73.1)	102 (68.9)	160 (79.2)	72 (67.3)	**0.03**	**0.02**
**Type of antibiotic prescribed before susceptibility testing**					0.8	0.9
Cefepime	36 (10.8)	9 (8.8)	21 (13.1)	6 (8.3)		
Ceftazidime	10 (2.9)	1 (1.0)	6 (3.8)	3 (4.2)		
Ciprofloxacin	36 (10.8)	12 (11.8)	17 (10.6)	7 (9.7)		
Imipenem	6 (1.8)	2 (2.0)	2 (1.3)	2 (2.8)		
Levofloxacin	6 (1.8)	3 (2.9)	2 (1.3)	1 (1.4)		
Meropenem	34 (10.2)	7 (6.9)	18 (11.3)	9 (12.5)		
Piperacillin/tazobactam	182 (39.8)	58 (56.9)	83 (51.9)	41 (56.9)		
Non antipseudomonal antibiotic	21 (6.27)	8 (7.8)	10 (6.3)	3 (4.2)		
Other	3 (1.1)	2 (2.0)	1 (0.6)	0 (0.)		
**Microbiologically documented coinfection**	179 (39.2)	59 (39.9)	81 (40.1)	39 (36.5)	0.8	0.5
**Type of antibiotic prescribed after susceptibility testing**					** < 0.001**	** < 0.001**
Cefepime	49 (10.7)	19 (12.8)	17 (8.4)	13 (12.2)		
Ceftazidime	31 (6.8)	8 (5.4)	13 (6.4)	10 (9.4)		
Ciprofloxacin	86 (18.8)	30 (20.3)	36 (17.8)	20 (18.7)		
Imipenem	3 (0.7)	1 (0.7)	1 (0.5)	1 (0.9)		
Levofloxacin	11 (2.4)	4 (2.7)	3 (1.5)	4 (3.7)		
Meropenem	65 (14.2)	5 (3.4)	51 (25.3)	9 (8.4)		
Piperacillin-tazobactam	206 (45.1)	77 (52.0)	80 (39.6)	49 (45.8)		
Other	6 (1.3)	4 (2.7)	1 (0.5)	1 (0.9)		
**IDs counselling after susceptibility testing**					** < 0.001**	**0.04**
None	217 (47.6)	88 (59.5)	78 (38.6)	52 (48.6)		
Continuation of empiric therapy	77 (16.8)	4 (2.7)	54 (26.7)	19 (17.8)		
Continuation of targeted therapy	49 (10.7)	23 (15.5)	16 (7.9)	10 (9.4)		
Start of antibiotic therapy	43 (9.4)	17 (11.5)	12 (5.9)	14 (13.1)		
Stop of antibiotic therapy	4 (0.9)	2 (1.4)	2 (0.9)	0 (0.0)		
Modification of empiric therapy	42 (9.2)	12 (8.1)	22 (10.9)	8 (7.5)		
Modification of targeted therapy	24 (5.3)	2 (1.4)	18 (8.9)	4 (3.7)		
**Dosing adjustment after susceptibility testing**	25 (10.4)	2 (2.7)	16 (14.4)	7 (12.7)	**0.03**	
**Outcomes**						
**Targeted antibiotic therapy**					0.1	
Continuation of empiric therapy	242 (52.9)	76 (51.4)	111 (54.2)	55 (51.4)		
Modification of empiric therapy	91 (19.8)	26 (17.6)	48 (23.7)	17 (15.9)		
Start of antibiotic therapy	125 (27.2)	46 (31.1)	43 (21.2)	36 (33.3)		
**Adequate targeted antibiotic dosing**	364 (80.4)	84 (57.9)	183 (91.1)	97 (90.7)	** < 0.001**	

* Patients with more than one immunosuppressive condition were counted once

** History of ESBL producing Enterobacteriaceae defined as a positive sample prior to the current episode of infection within the last six months

***Healthcare associated infections defined as vascular catheter-associated infections, catheter-associated urinary tract infections, ventilator-associated pneumonia, surgical site infections and infections occurring more than 48 h after admission to hospital

^**‡**^Empiric therapy was not initiated as physicians considered that due to patients’ conditions antibiotic prescription could be delayed until susceptibility testing results were available

The proportion of meropenem prescriptions after the susceptibility testing results have been made available varied during the three periods as followed: 3.4% (5/148) before EUCAST update, 25.3% (51/202) after EUCAST update without selective reporting and 8.4% (9/107) after EUCAST update with selective reporting (*P* < 0.001). The request for IDs consultations was higher in the second and third periods than before EUCAST update (40.5% (60/148) versus 61.4% (124/202) versus 51.4% (55/107), *P* =< *0.001*), as well as the use of increased dosages of non-carbapenems anti-pseudomonal antibiotics (57.9% (84/148) versus 91.1% (183/202) versus 90.7% (97/107), *P* = < *0.001*).

Compared with the reference period before EUCAST update (period 1), there was a significant difference in the odds of targeted meropenem prescriptions for patients included after EUCAST update without selective reporting (period 2) (OR 9.65, 95% CI [3.74–24.89]), and after EUCAST update with selective reporting (period 3) (OR 2.62 [0.85–8.07]), *P* < 0.001 (Table 2).

**Table 2:** Unadjusted risk factors associated with targeted meropenem prescription

	Overall (n = 457)	No meropenem prescription (n = 392)	Meropenem prescription (n = 65)	Univariate OR [95% CI]	*P-v*alue
**Periods of study**					** < 0.001**
Period #1 before EUCAST update	148 (32.4)	143 (96.6)	5 (3.4)	Ref	
Period #2 after EUCAST update w/o selective reporting	202 (44.2)	151 (74.8)	51 (25.3)	9.65 [3.74–24.89]	
Period #3 after EUCAST update with selective reporting	107 (23.4)	98 (91.6)	9 (8.4)	2.62 [0.85–8.07]	
**Age ≥ 65 years (%)**	265 (57.9)	229 (58.4)	36 (55.4)	0.88 [0.52–1.49]	0.6
**Female sex (%)**	176 (38.5)	154 (39.3)	22 (33.9)	0.79 [0.45–1.45]	0.4
**Immunosuppression (%)**	78 (17.1)	62 (15.8)	16 (24.6)	1.73 [0.92–3.25]	0.08
**History of ESBL infection/ colonisation (%)**	10 (2.2)	6 (1.5)	4 (6.2)	4.21 [1.15–15.38]	**0.03**
**Associated *P. aeruginosa* bacteraemia (%)**	63 (13.8)	52 (13.3)	11 (16.9)	1.33 [0.65–2.71]	0.8
**Sepsis or septic shock (%), missing = 2**	91 (20.0)	64 (16.4)	27 (41.5)	3.61 [2.06–6.34]	** < 0.001**
**Rapid or ultimately fatal disease (%)**	233 (50.9)	192 (48.9)	41 (63.1)	1.77 [1.03–3.05]	**0.03**
**Healthcare associated infection (%)**	256 (56.1)	210 (53.6)	46 (70.8)	2.09 [1.18–3.71]	**0.01**
**Gram-negative rod coinfection (%)**	117 (25.6)	93 (23.7)	24 (36.9)	1.88 [1.08–3.27]	**0.026**
**IDs consultation after susceptibility testing (%)**	240 (52.5)	204 (52.1)	36 (55.4)	1.14 [0.67–1.93]	0.6
**Low respiratory tract infection (%)**	176 (38.5)	139 (35.4)	37 (56.9)	2.40 [1.41–4.09]	**0.001**

*EUCAST* European Committee on Antimicrobial Susceptibility Testing,* ESBL* Extended-Spectrum Beta Lactamase producing Enterobacteriales,* IDs* Infectious Diseases specialist

Patients with a history of ESBL infection or colonisation (positive sample prior to the current episode of infection within the last six months), a sepsis or septic shock, a Gram-negative rod co-infection, a rapid or ultimately fatal disease, a low respiratory tract infection or a healthcare-associated infections, were also more likely to receive meropenem in unadjusted models.

## Discussion

The primary aim of EUCAST revised definitions of susceptibility test categories was to eliminate the ambiguity associated with the old “intermediate (I)” category [6]. The new “I” category represents a second susceptible category, defined as “susceptible – increased exposure”. However, this change was not always clear for clinicians who preferentially continued to select antibiotics reported as susceptible (S). Regarding wild-type *P. aeruginosa*, anti-pseudomonal antibiotics are now systematically reported as “I”, except for meropenem as the standard dosage allows reporting wild-type strains as “S”. We previously reported that the change to 2020 EUCAST criteria might be associated with an increase of meropenem prescriptions for the treatment of *P. aeruginosa* infections. These over-prescriptions were mainly switches of empiric therapy to meropenem after receiving the susceptibility testing results, stressing the need of prescribers' education and the importance of antibiotic stewardship interventions [2]. In the present study, we observed a decrease of meropenem prescriptions after the implementation of selective reporting, although the rates of meropenem prescriptions were still higher than in the first period (8.4% vs 3.4%). One explanation could be the higher rate of healthcare-associated infections in the third period compared to the first one which seems to be a risk of prescribing meropenem as empirical therapy without systematic de-escalation. It also highlights the fact that selective reporting should be associated with training and other antibiotic stewardship measures for clinicians.

The introduction of 2020 EUCAST criteria was also associated with a higher proportion of IDs consultations for *P. aeruginosa* infections in the second and third periods without and with selective reporting. These findings suggest that masking meropenem combined with infectious diseases consultations prevent over-prescription of meropenem.

Selective reporting of antibiotic susceptibility testing results has been described as a promising antibiotic stewardship tool to reduce inappropriate antibiotic prescriptions [4, 5, 7]. In the specific case of infections due to *P. aeruginosa,* this easy-to-implement strategy should be promoted to reduce meropenem over-prescriptions, regardless of the size of the hospital and the availability of IDs consultations.

As reported in our first study, we still observed a trend for higher use of adequate targeted antibiotic dosage for ceftazidime, cefepime and piperacillin-tazobactam after EUCAST criteria update (periods 2 and 3).

Our study has several limitations. First, it is a retrospective observational monocentric study with a small sample size and limited external validity. Second, since only 65 patients in total had a meropenem prescription after the susceptibility testing results have been made available, with only five prescriptions before the new EUCAST criteria were implemented, this did not allow us to perform a robust adjusted analysis. Hence, we cannot formally conclude that “selective reporting” was the main cause of reduction in meropenem prescriptions in the third period. Third, many IDs consultations are given orally and not documented in the EMR with a potential underestimation of IDs consultations and their impact on reducing inappropriate prescriptions. Furthermore, the three study periods were not equivalent in duration and in seasons with different epidemiological characteristics, which makes them not perfectly comparable. Another limitation is that we designed our study to evaluate the over-prescription of meropenem as a marker of the quality of care delivered. We did not collect and provide data on patient’s outcome. Finally, we have no data on phone calls to the microbiology laboratory to ask for meropenem susceptibility results if masked.

Despite these limitations, we observed a marked increase in meropenem prescriptions in our hospital after the implementation of the new EUCAST criteria, followed by a decrease after the setting up of the selective reporting. A learning effect and IDs consultations might also have played a role in this decrease. As mentioned by Turnidge et al., these results stress the importance of education and multimodal antibiotic stewardship strategies, as the selective reporting, to improve appropriate prescriptions after the introduction of revised definitions and breakpoint updates [6].

## Conclusion

In conclusion, multimodal antibiotic stewardship interventions with selective reporting of antibiotic susceptibility testing results for *Pseudomonas aeruginosa* and access to IDs consultations might have reduced the proportion of meropenem over-prescriptions after the 10th version of EUCAST breakpoint table updates in our hospital. These results suggest that selective reporting could be an interesting, easy-to-implement and cheap tool to reduce inappropriate broad-spectrum antibiotics prescriptions.

## Supplementary Information

**Additional file 1.** Supplementary figure 1.

## Data Availability

The datasets used and/or analysed during the current study are available from the corresponding author on reasonable request.

## Abbreviations

EUCAST: European Committee on Antimicrobial Susceptibility Testing
*P.** aeruginosa*: *Pseudomonas aeruginosa*
EMR: Electronic Medical Record
ESBL: Extended-spectrum beta-lactamase
IDs: Infectious diseases specialist
eCRF: Electronic clinical report form
SD: Standard deviation
IQR: Interquartile range
OR: Odds ratio
CI: Confidence interval
VD: Vaud
