# Supplementary figures and images for: Impact of selective reporting of antibiotic susceptibility testing results on meropenem prescriptions for the treatment of Pseudomonas aeruginosa infections after 2020 EUCAST criteria update: an observational study in a university hospital
Source: Antimicrob Resist Infect Control. 2022 Dec 30;11:165. doi: 10.1186/s13756-022-01203-x (PMC9805013; doi:10.1186/s13756-022-01203-x)

1336 Participants were assessed for eligibility

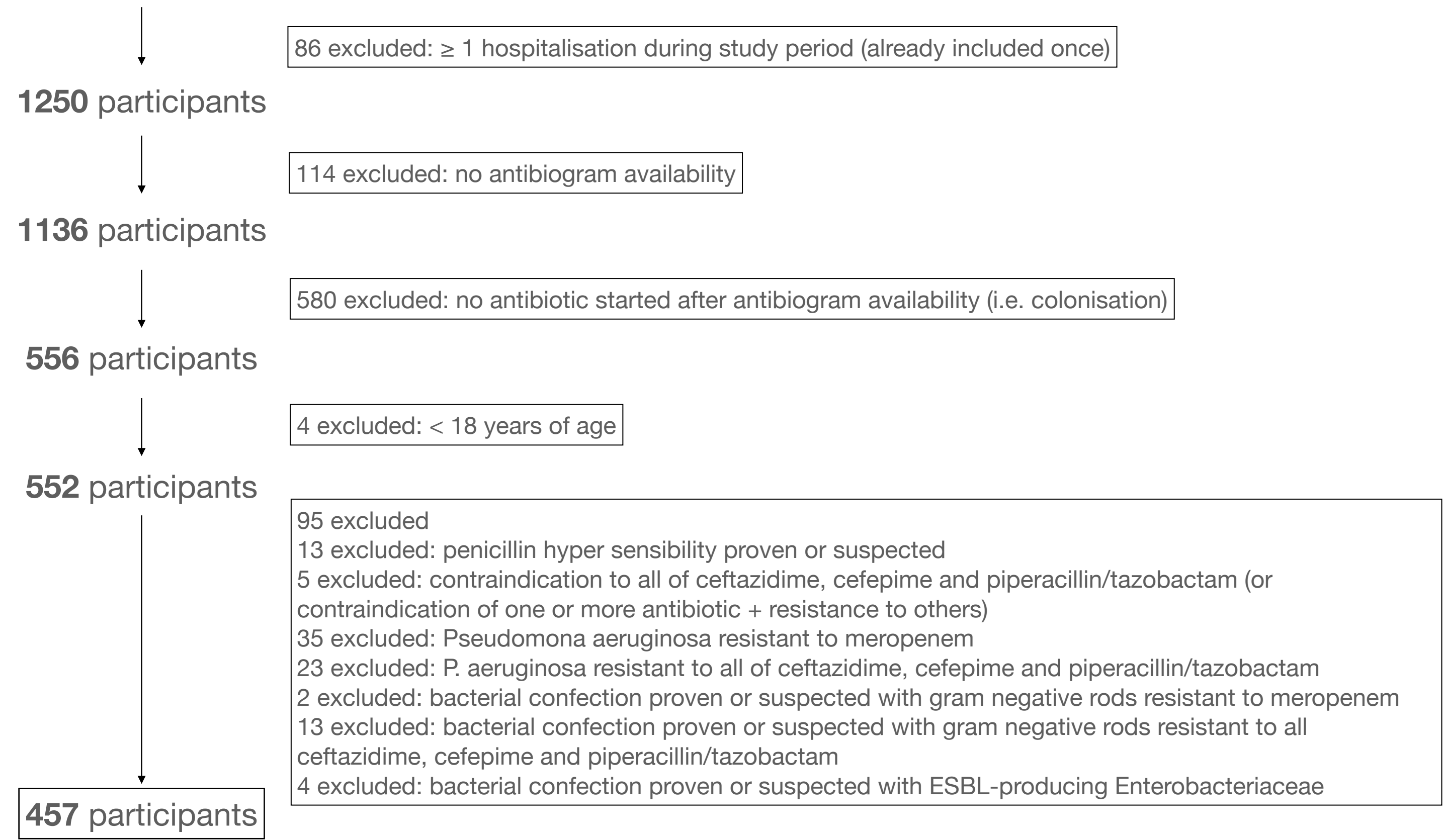

Supplement: Supplementary file 1 — Additional file 1. Supplementary figure 1. [file 13756_2022_1203_MOESM1_ESM.pdf]
